# Cellulose as a Delivery System of Raspberry Juice Volatiles and Their Stability

**DOI:** 10.3390/molecules25112624

**Published:** 2020-06-05

**Authors:** Josipa Vukoja, Anita Pichler, Ivana Ivić, Josip Šimunović, Mirela Kopjar

**Affiliations:** 1 Faculty of Food Technology, Josip Juraj Strosmayer University, F. Kuhača 18, 31000 Osijek, Croatia; jjosipa.vukoja@gmail.com (J.V.); anita.pichler@ptfos.hr (A.P.); ivana.ivic@ptfos.hr (I.I.); 2 Department of Food, Bioprocessing and Nutrition Sciences, North Carolina State University, Raleigh, NC 27695, USA; simun@ncsu.edu

**Keywords:** raspberry volatiles, cellulose, stability, encapsulation, freeze-drying, SPME, GC/MS analysis

## Abstract

Formulation of delivery systems for active ingredients is of increasing importance for the food industry. For that purpose, we selected cellulose as a carrier polymer of raspberry volatiles. Freeze-dried cellulose/raspberry complexes were prepared by complexation of raspberry juice (constant amount) and cellulose (2.5%, 5%, 7.5% and 10%). In our study, cellulose was shown as a good carrier of raspberry juice volatiles. Thirty-nine volatiles were detected in raspberry juice while 11 of them were lost during preparation of the complexes. Berry flavor note was the dominant one in raspberry juice (40% of overall flavor), followed by citrus and woody notes (each around 18% of overall flavor) and floral, fruity, and green (each around 8% of overall flavor). Cellulose/raspberry complexes had different flavor profiles, but a berry flavor note was still the dominant one in all complexes. These results suggest an efficient plant-based approach to produce value-added cellulose/volatile dry complexes with possible utility as food flavoring ingredients.

## 1. Introduction

Flavor is one of the most important quality attributes of fruit products composed of a complex mixture of volatile organic compounds [1]. Volatile organic compounds are organic molecules with appreciable vapor pressure at ordinary room temperature. They are usually small molecules with a molecular weight lower than 300 Daltons. 

People often associate scents with volatiles that can be perceived by the human nose and have a pleasant smell and flavor [2]. Raspberries (*Rubus idaeus* L.) are a member of the Rosaceae family which red fruits possess a sweet but tart flavor. Their typical flavor makes these fruits easily recognizable and appreciated [3] and it is caused by a complex combination of hundreds of volatile compounds [4]. The major classes of compounds include aliphatic and aromatic hydrocarbons, aldehydes, ketones, alcohols, esters, C13-norisoprenoids, monoterpenes and sesquiterpenes [5]. 

Flavor has become an essential characteristic for food developers. In the marketplace there is a growing demand for reduced use of artificial preparations thus natural flavor preparations are getting more attention in the food industry. Those preparations can contribute to a variety of foods, restore the flavoring lost during processing and allow the development of new products with novel tastes. Manufacturing processes, packaging, storage conditions and ingredients in foods often cause modifications and loss of flavor compounds and/or production of off-flavor components [6]. To limit flavor degradation or loss during processing and storage, it is beneficial to encapsulate volatile compounds prior to use in foods or beverages. Encapsulation refers to techniques by which a material is coated within another material forming a protective shell or wall [7,8]. 

The most common wall materials are carbohydrates (e.g., maltodextrins, modified starches, gum acacia etc.), proteins (e.g., gelatin or whey protein) or combinations of these materials. Several literature reviews detail the various encapsulation methods along with their respective strengths and weaknesses [9,10,11]. Encapsulation of flavors has been attempted and commercialized using many different methods such as spray drying, spray chilling or spray cooling, extrusion, freeze-drying, coacervation and molecular inclusion [8]. Freeze-drying is one of the most commonly used encapsulation techniques, especially for drying of thermosensitive substances that are unstable in aqueous solutions. 

The nature of delivery systems varies and it has a huge impact on the entrapment of volatiles during drying. This effect can be attributed to physical entrapment of volatiles within the matrix used (texture-specific effect) or to the binding to the polymer present in the matrix (agent-specific effect). As the result volatile molecule interactions with the matrix, which mainly involve binding, adsorption, complexation and encapsulation, are of critical importance, as well as the physicochemical properties of volatiles (like hydrophobicity, pressure, solubility and structure) [12,13,14,15,16]. Incorporation of small amounts of flavors into foods can influence the final quality of the product, cost, and consumer satisfaction. 

The food industry is continuously developing ingredients, processing methods, and packaging materials to improve flavor preservation and delivery [17]. The ability of carbohydrates to bind flavors is complemented by their diversity, low cost, and widespread use in foods and makes them the preferred choice for encapsulation [18,19]. Through drying, retention of volatiles on carbohydrates is a complex phenomenon that depends on several factors. These include the chemical and physical properties of the carbohydrates as well as the volatile compounds such as molecular weight and structure, stereochemistry and other characteristics which are related to different diffusion and sorption within the microstructure of the encapsulation material [15,20,21,22,23]. Retention of some native compounds after drying can be due to their low volatility. Also, since volatiles are generally larger than water molecules they may not readily diffuse or are trapped within the carbohydrate matrix during drying [23,24]. Process variables during freeze-drying also play an important role in the retention of volatiles on carbohydrate matrices. During freeze-drying the formation of microregions occurs and they become impermeable to organic compounds when their water content decreases below a critical level [24]. Several studies have investigated the effect of some types of carbohydrates like dextrin, starches, λ-carrageenan, β-cyclodextrins, xanthan, guar gum and pectins on the stability and retention of volatile compounds [25,26,27,28,29]. Cellulose is the polysaccharide composed of glucose molecules, and it is insoluble in water and common organic solvents. It is the main component of the plant cell walls, and widely distributed and abundant in nature. The structure and properties of cellulose make it a suitable material for the encapsulation of different active ingredients in order to formulate stable and efficient delivery systems [30,31,32,33,34,35,36], thus we choose it as a delivery system for raspberry volatiles. In addition, cellulose is one of the main dietary fibers. 

Generally, during the last several decades, dietary fibers have been the focus of many studies on their potential health benefits. Many epidemiological and clinical studies have demonstrated that the consumption of dietary fibers has positive effects on obesity, type 2 diabetes, cancer and cardiovascular disease [37]. The potential use of cellulose as a delivery system for flavor compounds was investigated in this study. In these complexes, the benefits of one plant compound would be combined with the benefits of other plant compounds producing an additive, which could then be used for development and/or improvement of novel, innovative foods. 

For that purpose, freeze-dried complexes of cellulose and raspberry volatiles were prepared. Complexation of cellulose and raspberry volatiles was conducted for 15 and 60 min in order to evaluate if prolonged complexation would result in a better adsorption of volatiles onto the cellulose. In addition, the amount of cellulose used was varied (2.5, 5, 7.5 and 10%). Adsorption of raspberry volatiles onto cellulose was investigated after the preparation of complexes, but additionally, the stability of the complexes during 12 months of storage was also investigated. 

## 2. Results

### 2.1. Evaluation of Volatiles

The results of total individual volatiles in raspberry juice and cellulose/raspberry complexes, together with the molecular weights, hydrophobicity, vapor pressure and odor description are presented in Table 1. 

Thirty-nine volatile compounds were identified in raspberry juice and thirty-five volatiles were identified in cellulose/raspberry complexes. The main classes were aldehydes (2-hexenal, octanal, 2-nonenal, nonanal, benzaldehyde, decanal, ethyl benzaldehyde, lilial, 4-propylbenzaldehyde, hexyl cinnamaldehyde, myristyl aldehyde), ketones (1-phenylethanone, geranyl acetone, benzophenone), terpenes (linalool, linalool oxide, menthol, nerol, geraniol, myrtenol, vitispirane, γ-terpinene α-terpineol, dihydro β-ionol, β-damascenone, α-ionol, α-ionone, dihydro-β-ionone, β-ionone, α-terpinolene, α-cedrol, *trans*-caryophyllene, β-myrcene, α-terpineol, myristicin, β-cyclocitral), alcohols (2-ethylhexanol, decanol, hexanol, octanol, benzyl alcohol), acids (ethyl hexanoic acid, nonanoic acid, hexanoic acid) and a phenol (guaiacol). 

**Figure 1:**
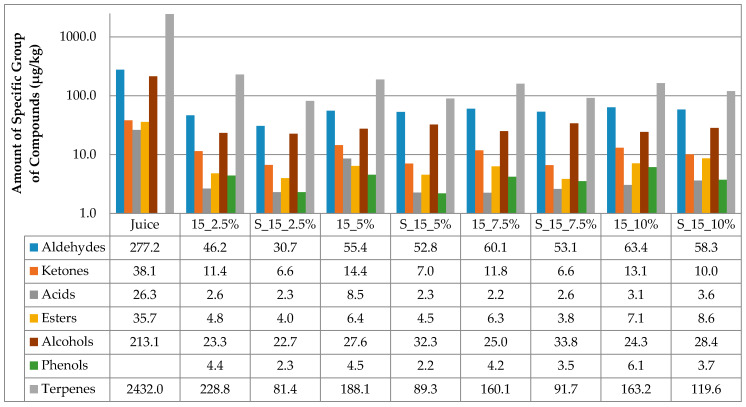
Amount of volatiles of specific chemical group in juice and on cellulose/raspberry complexes after 15 min of complexation and after storage (2.5%, 5%, 7.5% and 10%—amounts of used cellulose; 15—time of complexation; S—storage).

**Figure 2:**
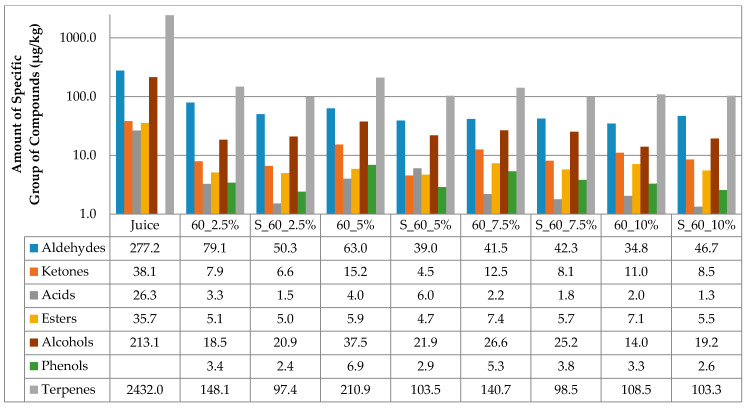
Amount of volatiles of specific chemical group in juice and on cellulose/raspberry complexes after 60 min of complexation and after storage (2.5%, 5%, 7.5% and 10%—amounts of used cellulose; 60—time of complexation; S—storage.

The results of the amount of volatiles of specific chemical groupa in juice and cellulose/raspberry complexes after complexation and after storage are presented at Figure 1 and Figure 2. Considering juice, terpenes were the most abundant class of volatile compounds, followed by aldehydes and alcohols, then ketones, esters and acids in the lowest amounts. 

Time of complexation and amount of cellulose had an impact on the amount of volatiles adsorbed onto cellulose. All cellulose/raspberry complexes, regardless of time of complexation and cellulose amount, also had terpenes as the most abundant class of volatile compounds, followed by aldehydes and alcohols. From the results, it can be observed that much lower amounts of volatiles were observed on cellulose/raspberry complexes then in juice. More detailed results of amount of each identified volatile compounds in juice and on cellulose/raspberry complexes are presented in Table 2. 

A few volatile compounds were not detected on cellulose/raspberry complexes (hexanol, octanol, γ-terpinene, vitispirane, benzyl alcohol, hexanoic acid, *trans*-caryophyllene, myristicin, myristyl aldehyde, β-myrcene, ethyl decanoate and β-cyclocitral) while a few new volatile compounds were detected (menthol, ethyl benzaldehyde, decanol, nonanoic acid, guaiacol, α-cedrol, benzophenone, hexyl salicylate). During the complexation and drying process, secondary compounds can be formed by various reactions in which the original fruit volatiles can be degraded.

From the results it can be seen that amount of volatiles highly depended on the amount of cellulose used and the time of complexation. Considering the aldehyde group, ten aldehydes were identified in cellulose/raspberry complexes, namely 2-hexenal, benzaldehyde, octanal, nonanal, 2-nonenal, decanal, ethyl benzaldehyde, 4-propylbenzaldehyde, lilial and hexyl cinnamaldehyde.

Among all aldehydes, decanal, with floral-like notes, was determined in the highest amount. Cellulose/raspberry complexes prepared with 7.5% of cellulose for 15 min had 25.98 µg/kg of decanal while complexes prepared with 2.5% using 60 min of complexation had the highest amount of this aldehyde (38.56 µg/ kg). Complexes of cellulose/raspberry prepared by 60 min of complexation with the addition of 5% of cellulose had the highest amount of benzaldehyde (8.45 µg/kg), nonanal (18.71 µg/kg), octanal (7.16 µg/kg), 4-propylbenzaldehyde (7.65 µg/kg) and lilial (3.31 µg/kg). Prolonged complexation had a high effect on adsorption of 2-hexenal and the highest amount (1.53 µg/kg) of this volatile was determined in complexes prepared with 2.5% of cellulose. 2-Nonenal was determined in the highest amount with 7.5% of cellulose (4.01 µg/kg), and ethyl benzaldehyde and hexyl cinnamaldehyde (5.25 µg/kg and 3.10 µg/kg, respectively) with 10% of cellulose prepared by 15 min of complexation.

There were three ketones identified in the cellulose/raspberry complexes and their behavior also depended on the time of complexation. All three of them were evaluated in the highest amount in complexes obtained with 5% of cellulose. A fruity flavor note ketone, 1-phenylethanone, was evaluated in the highest amount after 60 min of complexation, while two other floral flavor notes ketones, after 15 min of complexation (10.18 µg/kg and 2.91 µg/kg, respectively). Sixteen terpenes were detected in cellulose/raspberry complexes.

**Figure 3:**
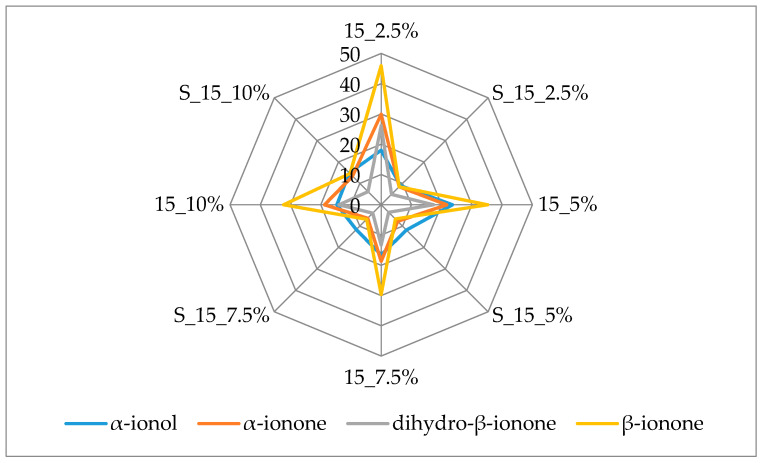
Amount of berry volatiles on cellulose/raspberry complexes after 15 min of complexation and after storage (2.5%, 5%, 7.5% and 10%—amounts of used cellulose; 15—time of complexation; S—storage).

**Figure 4:**
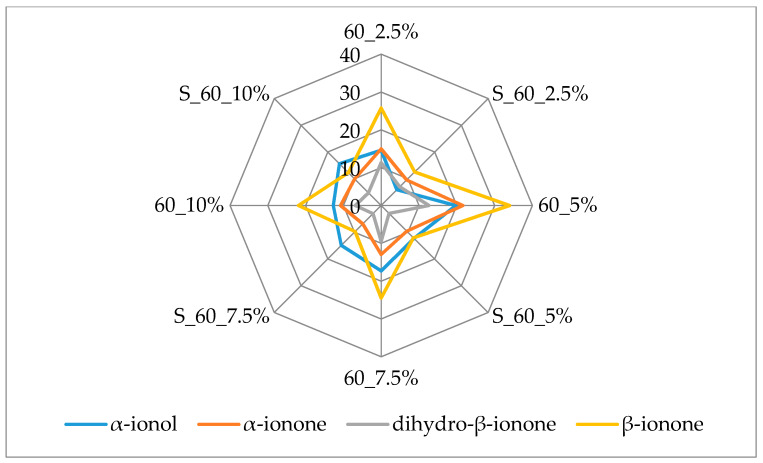
Amount of berry volatiles on cellulose/raspberry complexes after 60 min of complexation and after storage (2.5%, 5%, 7.5% and 10%—amounts of used cellulose; 60—time of complexation; S—storage).

Figure 3 and Figure 4 additionally present the variation of the most important terpenes responsible for the berry flavor note. β-Ionone and α-ionone, the most important raspberry flavor components [38,39] that produce the fruity, raspberry type flavor, displayed the highest amounts (45.90 µg/kg and 29.90 µg/kg, respectively) in the samples with 2.5% of cellulose prepared by 15 min complexation. Both of these volatiles were lost during prolonged complexation. 

The same behavior was observed for dihydro-β-ionone and α-ionol, volatiles with berry flavor notes. Dihydro-β-ionone had the highest amount in complex with 2.5% of cellulose (26.13 µg/ kg) and α-ionol in complex with 5% of cellulose (23.53 µg/ kg). The amount of cellulose used didn’t appear to have an influence on adsorption of dihydro-β-ionol after 15 min of complexation while after 60 min lower amounts of this compound were adsorbed on cellulose when it was used in higher amounts (7.5% and 10%) for complexation. β-Damascenone was evaluated in slightly higher amounts when 15 min of complexation was applied, but it was adsorbed in the highest amount with 5% of cellulose regardless of complexation time.

Linalool was determined in the highest amount among all identified terpenes. During 15 min of complexation with the increase of cellulose used a decrease of adsorption of this volatile occurred. 5% cellulose caused the highest adsorption (57.52 µg/kg) of linalool during 60 min of complexation. Adsorption of linalool oxide was quite high when cellulose was used in higher amounts (7.5% and 10%) for 15 min of complexation (around 5.6 µg/ kg). Prolonged complexation caused a loss of this volatile. Nerol, geraniol and α-terpinolene showed a similar behavior. Fifteen min of complexation caused the highest adsorption of these volatiles onto 2.5% of cellulose and when increased amounts of cellulose were used, a decrease of adsorption of these volatiles was observed.

On the other hand, through prolonged complexation the highest amount of these volatiles was observed with 5% of cellulose. Myrtenol was observed in the highest amount when a lower amount of cellulose was used, regardless of the time of complexation. Two alcohols were detected in cellulose/raspberry complexes, 2-ethylhexanol and decanol. The highest amount of 2-ethylhexanol, with a sweet, fruity flavor note, was in complexes prepared for 60 min with 5% of cellulose (35.09 µg/kg), and in this case prolonged complexation had quite a high impact on the adsorption of this volatile onto cellulose.

For the other alcohol, decanol, with fruity, citrus flavor notes, the time of complexation did not play such an important role and it was determined in the highest amount in complexes prepared for 15 min with 10% of cellulose (3.28 µg/kg). Also, two acids were found in complexes. Ethylhexanoic acid that was not found in the samples prepared by 60 min complexation, and the highest amount of nonanoic acid was seen in the samples prepared for 15 min with 5% of cellulose (8.12 µg/kg). Two esters, methyl dihydrojasmonate and hexyl salicylate, were also identified on the complexes. Methyl dihydrojasmonate was evaluated in higher amounts after 15 min of complexation while for hexyl salicylate, better results were achieved with prolonged complexation.

The stability of the obtained complexes was also evaluated during 12 months of storage. The amount of cellulose highly affected the retention of volatiles during storage. During storage (Figure 1 and Figure 2), lower amounts of specific chemical groups were observed in cellulose/raspberry complexes but the tendency was same as after preparation, i.e., terpenes were the most abundant class of volatile compounds, followed by aldehydes and alcohols. Even if terpenes were not found in the highest amount in complexes prepared with 10% of cellulose, their retention during storage was the highest in those complexes indicating their stability. Detailed results of individual volatiles in cellulose/raspberry complexes are given in Table 3.

Only 2-hexenal, the volatile with the lowest molecular weight, was completely lost during the storage in all complexes. Lilial was completely lost in the complexes prepared for 15 min, while in the complexes prepared by prolonged complexation this compound was quite stable.

Ethyl hexanoic acids had the reverse behavior of lilial, as it was completely lost in complexes prepared by prolonged complexation and stable in complexes prepared by 15 min complexation. Also, 1-phenylethanone was completely lost during storage conditions in the samples prepared for 60 min and it was retained only in the complex with 2.5% of cellulose prepared by 15 min of complexation.

There are variations in the retention of esters, alcohols, acids, ketones but it is quite possible that transformation reactions occurred during storage, thus changes of the ratios of these compounds is possible. For example, benzaldehyde was evaluated in much higher amounts, while the amount of ethyl benzaldehyde decreased throughout storage so it is possible that degradation of the latter compound occurred, resulting in the formation of benzaldehyde. 

Generally, terpenes are known for their very pleasant flavor notes responsible for the complete flavor profiles of fruits and they are quite desirable compounds. In all complexes regardless of complexation time, an overall higher retention of this volatile group was achieved with the addition of 7.5% and 10% of cellulose during complexation. From the results, it can be observed that preparation conditions (complexation time and cellulose amount) highly influenced the adsorption of volatiles and different ratio of volatiles were achieved. This ratio of volatiles that was achieved after preparation of complexes also affects stability of the compounds, i.e., losses during storage.

### 2.2. Comparison of Flavour Profile

**Figure 5:**
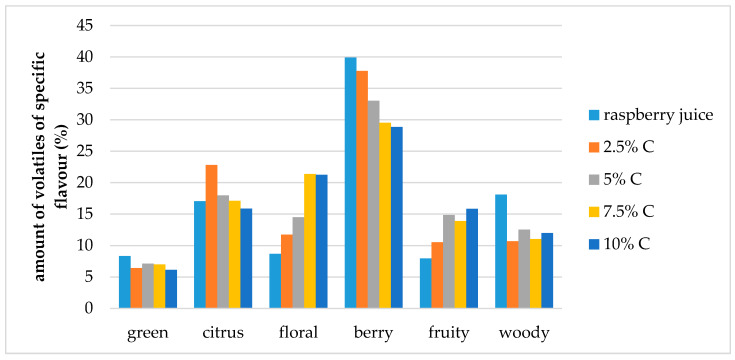
Amount of volatiles of specific flavor profile of raspberry juice and cellulose/raspberry complexes prepared by 15 min complexation (C—cellulose).

**Figure 6:**
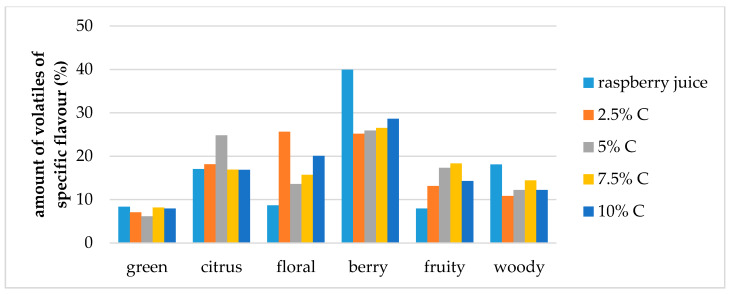
Amount of volatiles of specific flavor profile of raspberry juice and cellulose/raspberry complexes prepared by 60 min complexation (C—cellulose).

Carbohydrates have different abilities to entrap volatile compounds during drying processes so consequently this can change final flavor of the obtained dry products through changes in the retention of volatiles as well as their relative proportions [23,40,41,42,43,44]. Volatiles were divided into six different specific flavor note groups: green, citrus, fruity, floral, berry and woody which all combined together provide the overall flavor profile of raspberry. Comparison of the contribution of specific flavor notes to the overall flavor of raspberry juice and cellulose/raspberry complexes was conducted. Figure 5 and Figure 6 present these results.

Berry flavor (α-ionol, α-ionone, β-ionone, dihydro-β-ionone) note was the dominant one in raspberry juice (40% of overall flavor), followed by citrus and woody notes (each around 18% of the overall flavor) and floral, fruity and green (each around 8% of the overall flavor). Even though, cellulose/raspberry complexes had different flavor note profiles, the berry flavor note was still the dominant one in all complexes but its contribution to the complete flavor profile was lower.

In complexes prepared by 15 min of complexation berry flavor note contributed from 28% to 38% of the overall flavor (an increase was observed with a decrease of the cellulose amount) while in complexes prepared by 60 min of complexation, berry flavor note represented from 25% to 28% of the overall flavor.

The contribution of citrus flavor notes (nerol, limonene, linalool, nonanal) to the overall flavor profile was similar for complexes as for raspberry juice, with the exception of complexes with 2.5% and 5% cellulose for 15 and 60 min of complexation, respectively. Contribution to the overall flavor of those compounds in the mentioned complexes was around 24%.

Floral flavor note (decanal, lilial, geranyl acetone, benzophenone, geraniol, linalool oxide, hexyl cinnamaldehyde, methyl dihydrojasmonate, dihydro-β-ionol) was the highest in the samples prepared by 60 min of complexation with 2.5% of cellulose. With the amount of cellulose was increased from 2.5% to 10%, the amount of floral volatiles in the samples prepared for 15 min also increased.

Fruity flavor note (ethyl benzaldehyde, benzaldehyde, 2-ethylhexanol, 4 propylbenzaldehyde, ethyl decanoate) contributed to the overall flavor of complexes to a higher extent than to raspberry juice flavor. Woody flavor note (α-terpinolene, guaiacol, α-cedrol, α-terpineol, β-damascenone) contributed to the overall flavour of complexes to a lower extent and green (2-nonenal, menthol, octanal, myrtenol, hexyl salicylate) slightly lower than for raspberry juice.

**Figure 7:**
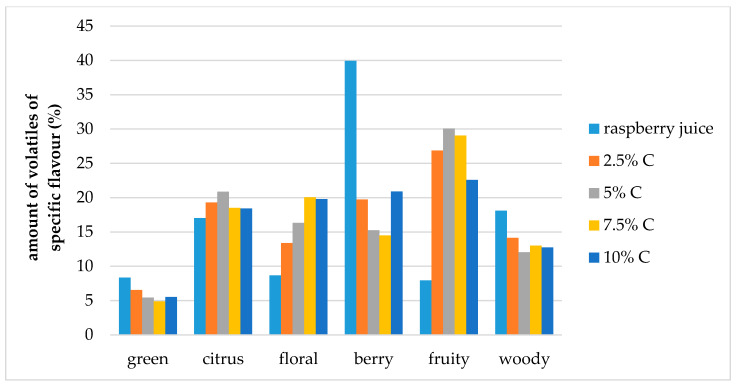
Amount of volatiles of specific flavor profile of raspberry juice and cellulose/raspberry complexes prepared by 15 min complexation (C—cellulose) after storage.

**Figure 8:**
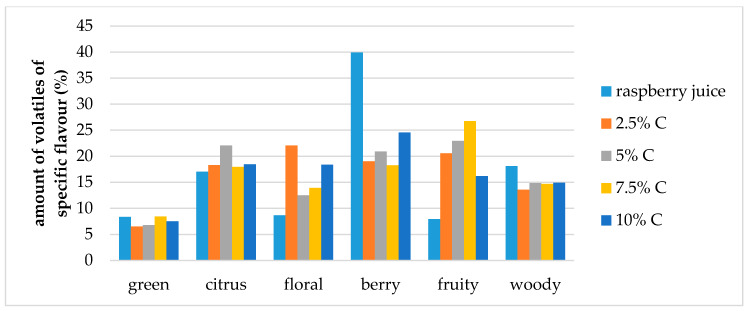
Amount of volatiles of specific flavor profile of raspberry juice and cellulose/raspberry complexes prepared by 60 min complexation (C—cellulose) after storage.

The effect of storage conditions on the volatile compound flavor profiles in the cellulose/raspberry complexes (prepared by 15 and 60 min complexation) after 12 months of storage is shown at Figure 7 and Figure 8. During storage, the ratios of specific flavor notes changed. Fruity flavor note became dominant in complexes, and berry, citrus and floral notes were balanced.

**Figure 9:**
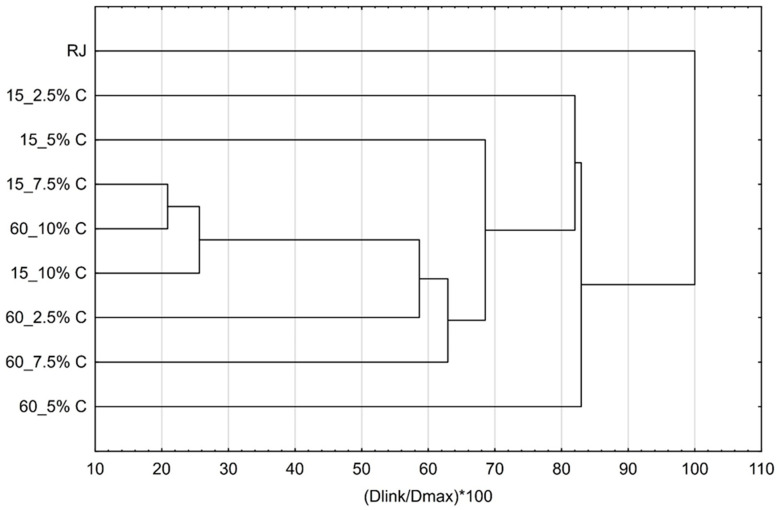
Cluster analysis of flavor profiles of cellulose/raspberry complexes after preparation.

**Figure 10:**
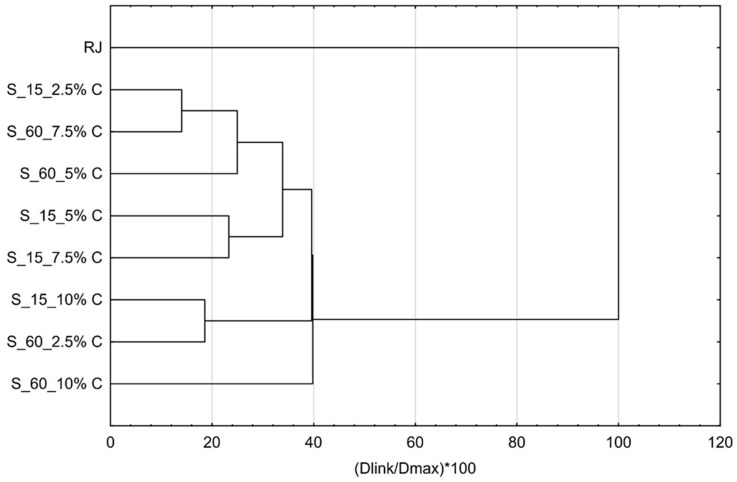
Cluster analysis of flavor profiles of cellulose/raspberry complexes after storage.

Additionally, cluster analysis of flavor profiles was conducted to evaluate which of the complexes are the most similar to each other and to raspberry juice after preparation and storage. Results are presented by Figure 9 and Figure 10. The most similar complexes were obtained by adsorption of raspberry volatiles onto 7.5% of cellulose for 15 min and 10% of cellulose for 60 min. One more complex was similar to those two; it was prepared with 10% of cellulose for 15 min. All the other complexes highly differ from those. The most similar complex to raspberry juice was the complex prepared by adsorption of raspberry volatiles onto 5% of cellulose for 60 min.

During storage, the flavor profiles changed and there was a quite high difference between the raspberry juice profile and cellulose/raspberry complex profiles. Complexes were grouped into three main categories. First one was the complexes obtained by adsorption of raspberry volatile onto 7.5% of cellulose for 60 min and 2.5% of cellulose for 15 min, the second one was complexes obtained after 15 min of complexation with 5% and 7.5% of cellulose and third group were complexes made with 2.5% of cellulose for 60 min and 10% of cellulose for 15 min. These results showed that storage stability of complexes regarding flavor profile highly depended on the cellulose amount and time of complexation.

## 3. Discussion

Formulation of delivery systems of active ingredients, exemplified in our study by volatile compounds, is of increasing importance for the food industry. Encapsulation systems should be carefully selected, usually due to the high sensitivity of active ingredients. Amorphous carbohydrates have been identified as very effective encapsulation matrices for delivering active ingredients since they can reduce the rate of release of flavor during storage and minimize the rate of oxidation of oxygen-sensitive flavors by environmental oxygen. In both cases the diffusion of guest molecules through the carbohydrate matrix is involved [45].

Retention and stability of volatiles is a complex phenomenon that depends on many factors such as the physicochemical properties of the volatile compounds, as well as type and concentration of the carbohydrate used [15,20,21,22,46]. Volatiles with high molecular weight have higher retention than low molecular weight volatiles in carbohydrates matrix [47]. Rosenberg et al. [48] showed that retention increased with the molecular weight of the volatile compounds when they studied esters which were spray-dried with gum arabica.

It was detected that ethyl hexanoate with higher molecular weight had a higher retention than ethyl butyrate. These results were explained by the greater ability of low molecular weight compounds to diffuse through the matrix during the drying process. Indeed, since these molecules are not linear, molecular weight and molecular size are linked and the latter is the primary factor determining the diffusion of components.

When the molecular weight of the volatile increases, its molecular size increases which slows down its diffusion rate [47]. As we observed in our research, not all volatiles of raspberry juice bonded with cellulose during the complexation process, as some of them (such as hexanol, octanol, γ-terpinene, vitispirane, benzyl alcohol, hexanoic acid, trans-caryophyllene, myristicin, myristyl aldehyde and β-cyclocitral) were not detected in cellulose/raspberry complexes. Those volatiles have either low molecular weight or low vapor pressure or a combination of these properties. The same properties were also observed for volatiles that were lost during storage (2-hexenal, lilial, 1-phenyl-ethanone).

There is a relationship between chemical groups of several classes of flavor compounds and their retention rates. Several studies [26,46,49,50] have showed that alcohols are usually the best retained compounds by carbohydrates because of glycoside linkages with carbohydrate [26]. Kim and Maga [49] studied the retention of volatile compounds (acids, aldehydes and alcohols) in high amylose starch and observed that alcohols had the greatest and aldehydes the lowest retention. A study on the influence of λ-carrageenan on volatile compounds (aldehyde, ketones, ester and alcohol) showed that esters had the highest volatility, followed by aldehydes, ketones and with alcohols as the lowest [50].

Similar results were obtained for the inclusion efficiency of watermelon flavor by γ-cyclodextrin which decreased in the order of alcohols > aldehydes > esters [46]. When compared to the previous studies about linkage between carbohydrates and chemical groups of volatiles, our results are partially in agreement. As already mentioned, during complexation as well as storage some aldehydes were lost, but also two alcohols from raspberry juice didn’t bind to cellulose, thereby showing the importance of chemical structure and the properties of the carrier.

The hydrophobicity of volatile compounds is also an important factor that affects retention and release of flavor. Retention of polar (hydrophilic) compounds is expected to be very low. Terta et al. [51] studied the retention of limonene and *trans*-2-hexenal in gum Arabica and propylene glycol alginate solutions by gas chromatography. Limonene, with the higher hydrophobicity, had higher retention than *trans*-2-hexanal which is quite polar. Polar (hydrophilic) compounds are more soluble in water and can diffuse more easily through the matrix, which can explain the lower retention of polar volatile compounds [52].

Terpenes that were generally better retained throughout storage on the complexes with higher amount of cellulose, have higher molecular weight in combination with high values of hydrophobicity. Release ratio of watermelon flavor from γ-cyclodextrin inclusion complexes decreased with the increase of hydrophobicity, implying the different affinities of hydrophobic flavor guests to γ-cyclodextrin [46]. This could slow down diffusion of volatiles during the storage period.

Structure of carbohydrate is very important, and usually it is not sufficient for the polymer to be able to bind volatiles, but some other properties may also be required. Investigation on the binding of different volatiles to β-cyclodextrin and α-cyclodextrin revealed the importance of carrier structure. Investigated volatiles had higher affinity for β-cyclodextrin due to the different size of cyclodextrin cavity.

Additionally, the hydrophobicity of volatiles was also important, proving that the driving force for complex formation was hydrophobic/hydrophilic interactions [53]. Oxidized corn starch and oxidized amaranth starch were used for encapsulation of vanillin as a possible replacement of gum arabica. It was observed that oxidized starch forms can be used for encapsulation of this compound with similar efficiency as gum arabica and additional advantages, such as freedom from hygroscopicity [54]. Application of cellulose nanocrystals in preparation of starch-based film structures caused change in permeability of d-limonene [55]. Encapsulating agents such as maltodextrins, modified starch, gum arabica, xanthan gum and β-cyclodextrin were used for encapsulation of synthetic strawberry flavor by different drying techniques. The results revealed that the blend of maltodextrins/modified starch/β-cyclodextrin exhibited the best capability for encapsulating the flavor, followed by maltodextrins/gum arabica/β-cyclodextrin and maltodextrins/xanthan gum/β-cyclodextrin [56,57].

Flink and Karel [25] studied the retention of organic volatiles in freeze-dried solutions containing various carbohydrates. Based on the experiments and studies on freeze-dried samples, a mechanism for the retention and loss of volatiles during drying was postulated. The mechanism involves formation of micro-regions in the dry cake in which volatiles are entrapped. The bulk structure of the dry cake indicates that these micro-regions are held together in some type of matrix, most likely made up of bridges of H-bonded carbohydrates. These micro-regions become impermeable to organic compounds when the water content decreases below a critical level. Their experiments have shown that slow freezing and decreasing sample thickness promoted retention of volatiles. An increase in polysaccharide concentration leads to decrease in the release of flavor compounds due to complexation and viscosity effect of the polysaccharide themselves.

Secouard et al. [15] studied the release of limonene and menthol from different xanthan solutions. They found out that with higher xanthan concentration, the release of limonene decreased which was explained by the higher hydrophobic character of xanthan in comparison to other hydrocolloids. Xanthan consists of a cellulose backbone with ionized trisaccharide branches. The ordered molecule is stabilized through hydrogen bonds by non-covalent side chain–main chain interactions involving hydrogen bonding. In this way, xanthan may create a hydrophobic interior in the carbohydrate molecule, which can include volatile compounds.

The hydrophobic character of cellulose, properties of volatiles and interactions between them during complexation as well as the formation of micro-regions during freeze-drying could be the combination that makes cellulose an acceptable carrier of raspberry volatiles. Decreases, increases or no effect on the amount of volatiles with the increase of amount of used cellulose were observed. However, for some compounds no regular tendency was observed. It is not unlikely that the various compounds compete for binding sites when they are added as a mixture of compounds [43,58,59,60]. In our research the amount of initial volatiles was constant but the amount of cellulose changed, probably having an impact on the number of available sites for interacting with volatiles.

As was already mentioned, cellulose is composed of glucose units bonded with β-d-(1-4) linkages and linked with intermolecular hydrogen bonds, and these bonds are responsible for cellulose crystalline packing and its properties [30,61]. The complete cellulose structure has an effect on causing van der Waals or hydrogen interactions. Hydroxyl groups of cellulose are linked to a glucopyranose ring, forming a hydrophilic state parallel to the ring plane causing hydrogen bond formation. Also, the -CH- groups are linked to the glucopyranose rings axially and this causes formation of a hydrophobic part perpendicular to the rings causing van der Waals interactions [62]. Probably, both van der Waals interactions and hydrogen bonding were involved in the interactions between cellulose and raspberry juice volatiles.

Additionally, the source of raspberry volatiles was juice, which is composed of both volatile and non-volatile compounds, i.e., it is complex matrix of different compounds thus other compounds could play a role in the complexation of volatiles with cellulose. Other studies also showed different behavior of volatiles depending on changes in the amount of carbohydrates. An investigation on the impact of sucrose, maltose and trehalose amount (5, 10 and 20%) on benzyl alcohol, benzaldehyde and 2-hexenal also showed an irregular behavior during freeze-drying [63]. A study on the behavior of sour cherry puree volatiles and the flavor profiles of obtained samples depended on the used sugars and their amount, starting with the same amount of volatiles [64].

## 4. Materials and Methods

### 4.1. Materials

Cellulose (microcrystalline) was obtained from Kemika (Zagreb, Croatia) and eugenol was obtained from Sigma (Darmstadt, Germany).

### 4.2. Preparation of Cellulose/Raspberry Complexes

Raspberry juice was prepared by pressing the raspberry fruit and filtration through cheesecloth. After that, raspberry juice was heated at 90 °C for 3 min. For the preparation of cellulose/raspberry complexes, cellulose and raspberry juice were mixed using a magnetic stirrer for 15 or 60 min at room temperature. Obtained mixtures were centrifuged for 15 min at 4000 rpm. After that, the liquid part was separated from the precipitate and precipitate was freeze-dried to obtain dry powder. Prior to freeze-drying precipitate was frozen at −18 °C for 24 h and then freeze-dried in a laboratory freeze-dryer (Christ Freeze Dryer, Alpha 1-4, Osterode am Harz, Germany) under the following conditions: freezing temperature was adjusted at −55 °C; the temperature of sublimation from −35 °C to 0 °C; and the vacuum level 0.220 mbar. The temperature of the isothermal desorption varied from 0 °C to 21 °C under the vacuum of 0.060 mbar. Complete process lasted for 12 h. One part of the obtained freeze-dried complexes were evaluated for volatile compounds while the other part of complexes were packed in sealed plastic bags and stored at room temperature, exposed to light for 12 months.

### 4.3. Volatile Compounds Analysis

Evaluation of the amounts of volatile compounds was conducted by gas chromatography/mass spectrometry (GC/MS) analysis after their extraction. Extraction of volatile compounds from the dried complexes was conducted using solid-phase microextraction (SPME). For that purpose, 0.3 g of sample was weighed into a 10 mL glass vial followed by the addition of 4.7 g of water and 1 g of NaCl. For the extraction of volatiles, SPME fiber coated with divinylbenzene/ carboxen/polydimethylsiloxane (DVB/CAR/PDMS) sorbent (50/30 μm, StableFlex™, Supelco, Bellefonte, PA, USA) was used.

Conditioning of the sample vials was performed in a temperature-controlled heating module at 45 °C for 45 min and agitated at 350 rpm. After extraction, fiber was removed from samples and volatiles were thermally desorbed in the injector port of the GC. Analysis was conducted on a GC 7890B gas chromatograph (Agilent Technologies, Santa Clara, CA, USA) equipped with a 5977A mass spectrometer (Agilent Technologies). Volatile compounds were desorbed into a GC injector port at 250 °C for 7 min; splitless mode. The gas chromatograph was fitted with a HP5 capillary column (60 m × 0.25 mm × 0.25 μm). Helium was used as the carrier gas at a flow rate of 1 mL/min at 40 °C. Oven temperature was programmed as follows: the initial temperature of 40 °C was held for 2 min, then 6 °C/min up to 230 °C.

The volatile compounds were identified using a mass selective detector. The detector operated in the *m/z* range between 45 and 450, ion source and quadrupole temperature were maintained at 230 and 150 °C, respectively. Compounds were confirmed by matching their mass spectra with the National Institute of Standards and Technology mass spectral database (NIST, East Amwell Township, NJ, USA) and through retention time (RT) and retention index (RI) (Table 1). Two repetitions were conducted for each sample. Quantification was conducted by eugenol as an internal standard and results were presented as µg/kg.

### 4.4. Statistical Analysis

Results were expressed as the mean values ± standard deviation. Data of volatile compounds amount were compared by analysis of variance (ANOVA) and Fisher’s least significant difference (LSD) with the significance defined at *P* < 0.05. In addition, after dividing volatiles into the groups by characteristic flavour profile, cluster analysis was conducted. For that purpose, joining (tree cluster) mode was used with single linkage amalgamation rule. All statistical analyses were carried out using software program STATISTICA 13.1 (StatSoft Inc., Tulsa, OK, USA).

## 5. Conclusions

Potential use of cellulose as a delivery system for raspberry flavor compounds was investigated in order to produce an additive which can be used for development and/or improvement of novel, innovative foods. Throughout our research we observed that amount of cellulose used as carrier polymer for volatiles as well as complexation time had an influence on the adsorption efficiency. The most important volatiles of raspberry are those contributing to the berry flavor note and through complexation with cellulose we achieved their adsorption onto polymer, retaining the berry flavor note as the dominant one in the produced complexes. The most efficient complex regarding those compounds was prepared by 15 min of complexation with 2.5% of cellulose and with an increase of the cellulose amount a decrease of adsorption of those volatiles was observed. On the other hand, the highest stability of these volatiles during storage was observed in complexes prepared with 10% of cellulose. By combining plant compounds that differ in their benefits, we achieved an efficient plant-based approach to produce value-added cellulose/volatile dry complexes with possible utility as flavoring food ingredients.

## Figures and Tables

**Table 1:** Volatile compounds detected in raspberry juice and cellulose/raspberry complexes.

Juice	Complex *	RT (min) ^1^	RI ^2^	MW ^3^	log P (o/w) ^4^	Vapor Pressure (mm/Hg)	Odor Description
**2-hexenal**	+	7.4716	851	98.14	1.79	4.62	green
**hexanol**	-	8.5358	866	102.18	2.03	0.947	green
**benzaldehyde**	+	14.8880	955	106.124	1.480	1.27	fruity
**β-myrcene**	-	17.1058	987	136.238	4.17	2.29	woody
**octanal**	+	18.0072	998	128.22	2.951	2.068	green
**hexanoic acid**	-	18.7302	1009	116.16	1.920	0.158	fruity
**limonene**	+	19.3232	1018	136.238	4.57	0.198	citrus
**2-ethyl hexanol**	+	19.8268	1029	130.231	2.82	0.207	fruity
**benzyl alcohol**	-	20.1599	1035	108.139	1.100	0.094	fruity
**γ-terpinene**	-	21.1997	1051	136.238	4.5	1.075	citrus
**1-phenylethanone**	+	21.6789	1057	120.151	1.58	0.397	fruity
**linalool oxide**	+	22.0851	1065	170.252	1.375	0.002	floral
**octanol**	-	22.2638	1071	130.23	3.0	0.079	green
**-**	guaiacol	23.1329	1080	124.139	1.32	0.179	woody
**α-terpinolene**	+	23.8974	1094	136.238	4.470	1.126	woody
**linalool**	+	23.8884	1096	154.252	2.970	0.016	citrus
**nonanal**	+	24.1158	1095	142.242	3.461	0.532	citrus
**ethyl hexanoic acid**	+	25.8217	1128	144.214	2.640	0.03	no flavor
**2-nonenal**	+	27.0726	1155	140.226	3.319	0.256	green
**-**	menthol	27.6494	1167	156.27	3.216	0.032	green
**α-terpineol**	+	28.4620	1180	154.253	2.67	0.028	woody
**myrtenol**	+	28.7553	1185	152.24	3.22	0.018	green
**decanal**	+	29.4771	1200	156.269	3.97	0.207	floral
	ethyl benzaldehyde	29.672	1201	134.178	2.408	0.106	fruity
**β-cyclocitral**	-	29.9239	1207	152.237	3.10	0.176	herbal
**nerol**	+	30.5818	1218	154.253	3.47	0.013	citrus
**geraniol**	+	31.9384	1247	154.253	3.56	0.021	floral
**4-propyl benzaldehyde**	+	32.4908	1261	148.20	2.918	0.039	no flavor
**vitispirane**	-	32.7283	1265	192.302	3.62	0.022	floral
**-**	decanol	32.7995	1265	158.28	4.570	0.008510	fruity
**-**	nonanoic acid	33.5062	1277	158.23	3.42	0.009	waxy
**α-ionol**	+	37.4621	1376	194.317	4.492	0.001	berry
**β-damascenone**	+	37.6571	1380	190.286	4.04	0.02	woody
**ethyl decanoate**	+	38.0311	1389	200.322	4.861	0.034	fruity
***trans*-caryophyllene**	-	38.5426	1402	204.356	6.777	0.013	woody
**α-ionone**	+	38.9487	1417	192.302	3.995	0.014	berry
**dihydro β-ionone**	+	39.2249	1432	194.317	3.990	0.01	berry
**dihydro-β-ionol**	+	39.4117	1440	196.33	4.634	0.001	floral
**geranyl acetone**	+	39.6066	1448	194.318	3.834	0.016	floral
**β-ionone**	+	4.3458	1477	192.302	3.995	0.017	berry
**myristicin**	-	41.0120	1511	192.214	2.586	0.008	woody
**lilial**	+	41.0691	1514	204.313	4.216	0.005	floral
**-**	α-cedrol	42.4579	1592	222.372	4.33	0.001	woody
**myristyl aldehyde**	-	42.4825	1601	212.376	6.008	0.006	woody
**-**	benzophenone	42.799	1618	182.222	3.1	0.001	floral
**methyl dihydrojasmonate**	+	43.1727	1647	226.316	2.653	0.001	floral
**-**	hexyl salicylate	43.4814	1667	222.284	5.07	0.00049	green
**hexyl cinnamaldehyde**	+	44.3590	1737	216.324	4.866	0.001	floral

^1^ RT—retention time of volatiles; ^2^ RI—retention index of volatiles; ^3^ MW—molecular weight; ^4^ log P—logarithm of octanol water coefficient that indicates the relative hydrophobicity of compound; data were obtained from http://www.thegoodscentscompany.com and http://www.chemicalbook.com; * detected ”+” and not detected “-“volatile compounds in juice and cellulose/raspberry complexes.

**Table 2:** Volatile compounds (µg/kg) of juice and cellulose/raspberry complexes prepared by 15 min and 60 min of complexation (2.5%, 5%, 7.5% and 10%—amounts of used cellulose).

Compounds	Juice	2.5%		5%		7.5%		10%	
Time of Complexation	-	15	60	15	60	15	60	15	60
**Aldehydes**									
**2-hexenal**	37.44 ± 0.05 ^a^	0.22 ± 0.00 ^d^	1.53 ± 0.05 ^b^	0.20 ± 0.03 ^d^	0.57 ± 0.08 ^c^	-	0.57 ± 0.03 ^c^	0.22 ± 0.04 ^d^	0.25 ± 0.04 ^d^
**benzaldehyde**	42.03 ± 1.17 ^a^	1.48 ± 0.00 ^f^	4.58 ± 0.47 ^c^	4.84 ± 0.87 ^c^	8.45 ± 0.99 ^b^	2.06 ± 0.07 ^e^	4.97 ± 0.31 ^c^	2.20 ± 0.40 ^d,e^	2.98 ± 0.32 ^d^
**octanal**	12.37 ± 0.39 ^a^	6.60 ± 0.41 ^b^	3.75 ± 0.24 ^d^	6.56 ± 1.21 ^b^	7.16 ± 1.56 ^b^	3.54 ± 0.09 ^d^	4.08 ± 0.13 ^c^	3.39 ± 0.30 ^d^	3.43 ± 1.23 ^d^
**nonanal**	27.63 ± 0.03 ^a^	17.02 ± 1.71 ^b^	12.16 ± 0.13 ^c^	17.16 ± 2.60 ^b^	18.71 ± 1.70 ^b^	11.05 ± 1.00 ^c,d^	11.78 ± 0.33 ^c,d^	11.44 ± 0.12 ^d^	8.59 ± 2.46 ^e^
**2-nonenal**	4.77 ± 0.12 ^a^	1.16 ± 0.01 ^d^	1.02 ± 0.08 ^d^	2.33 ± 0.13 ^c^	2.45 ± 0.28 ^c^	4.01 ± 0.02 ^b^	1.28 ± 0.16 ^d^	1.24 ± 0.06 ^d^	0.88 ± 0.13 ^e^
**decanal**	39.56 ± 2.80 ^a^	9.75 ± 1.12 ^c^	38.56 ± 2.40 ^a^	10.07 ± 1.55 ^c^	10.78 ± 0.87 ^c,d^	25.98 ± 2.32 ^b^	8.19 ± 0.98 ^d^	22.00 ± 2.80 ^b^	9.59 ± 0.50 ^c^
**ethyl benzaldehyde**	-	1.19 ± 0.08 ^d^	4.38 ± 0.42 ^b^	1.77 ± 0.31 ^c^	1.31 ± 0.51 ^c^	4.72 ± 0.11 ^b^	1.12 ± 0.12 ^d^	5.25 ± 0.01 ^a^	1.02 ± 0.32 ^d^
**4-propyl benzaldehyde**	92.06 ± 0.58 ^a^	4.15 ± 0.36 ^d^	7.44 ± 0.90 ^c^	7.50 ± 0.86 ^c^	7.65 ± 1.28 ^c^	4.21 ± 0.59 ^d^	4.43 ± 0.08 ^d^	11.30 ± 0.73 ^b^	2.89 ± 0.27 ^e^
**lilial**	8.64 ± 0.03 ^a^	2.74 ± 0.05 ^c^	2.84 ± 0.73 ^b,c^	2.77 ± 0.35 ^b,c^	3.31 ± 0.50 ^b^	2.35 ± 0.30 ^c^	2.61 ± 0.34 ^b,c^	3.30 ± 0.46 ^b^	2.34 ± 0.90 ^b,c^
**hexyl cinnamaldehyde**	4.07 ± 0.12 ^a^	1.91 ± 0.03 ^f^	2.81 ± 0.06 ^b,c^	2.22 ± 0.05 ^d^	2.65 ± 0.13 ^c,e^	2.14 ± 0.29 ^d,f^	2.44 ± 0.10 ^d,e^	3.10 ± 0.26 ^b^	2.82 ± 0.48 ^b,c^
**Ketones**									
**1-phenylethanone**	9.30 ± 0.18 ^a^	3.53 ± 0.24 ^c^	1.02 ± 0.04 ^f^	2.19 ± 0.16 ^d^	4.43 ± 0.12 ^b^	1.71 ± 0.48 ^e^	1.35 ± 0.15 ^e^	2.27 ± 0.18 ^d^	0.94 ± 0.32 ^f^
**geranyl acetone**	28.77 ± 0.10 ^a^	6.29 ± 0.49 ^d,e^	6.89 ± 1.22 ^d,f^	10.18 ± 1.41 ^b^	8.94 ± 1.46 ^b,c,f^	8.26 ± 0.72 ^b,c,f^	7.60 ± 0.15 ^c,f^	7.87 ± 0.93 ^c,f^	7.70 ± 1.26 ^c,e,f^
**benzophenone**	-	1.61 ± 0.13 ^e^	2.52 ± 0.50 ^b,c^	2.05 ± 0.07 ^d^	1.82 ± 0.41 ^d,e^	1.79 ± 0.45 ^d,e^	3.54 ± 0.39 ^a^	2.91 ± 0.40 ^a,b^	2.39 ± 0.47 ^c^
**Esters**									
**methyl dihydrojasmonate**	6.94 ± 0.08 ^a^	1.63 ± 0.21 ^d^	3.06 ± 0.08 ^d^	3.33 ± 0.38 ^c^	3.23 ± 0.06 ^c^	1.41 ± 0.12 ^a^	4.59 ± 0.26 ^b^	3.44 ± 0.38 ^c^	2.73 ± 0.66 ^c^
**hexyl salicylate**	-	3.17 ± 0.03 ^b^	2.05 ± 0.27 ^c^	3.08 ± 0.26 ^b^	2.63 ± 0.27 ^c^	4.91 ± 0.84 ^a^	2.76 ± 0.52 ^a^	3.66 ± 0.65 ^a^	4.39 ± 0.67 ^a^
**Acids**									
**hexanoic acid**	22.44 ± 0.42	-	-	-	-	-	-	-	-
**ethyl hexanoic acid**	3.84 ± 0.28 ^a^	1.22 ± 0.12 ^b^	-	0.41 ± 0.04 ^d^	-	0.72 ± 0.08 ^c^	-	0.76 ± 0.30 ^c^	-
**nonanoic acid**	-	1.41 ± 0.13 ^f^	3.26 ± 0.45 ^c^	8.12 ± 0.94 ^a^	4.00 ± 0.06 ^b^	1.51 ± 0.44 ^e,f^	2.19 ± 0.14 ^d^	2.29 ± 0.09 ^d^	2.04 ± 0.25 ^d,e^
**Alcohols**									
**hexanol**	83.19 ± 0.43	-	-	-	-	-	-	-	-
**2-ethyl hexanol**	37.99 ± 2.88 ^a^	21.21 ± 0.48 ^b^	16.07 ± 0.20 ^c^	25.21 ± 4.33 ^b^	35.09 ± 2.76 ^a^	22.06 ± 0.55 ^b^	24.56 ± 0.13 ^b^	20.98 ± 2.23 ^b^	11.98 ± 0.77 ^d^
**benzyl alcohol**	10.12 ± 0.02	-	^-^	-	-	-	^-^	-	^-^
**octanol**	81.78 ± 2.57	-	-	-	-	-	-	-	-
**decanol**	-	2.12 ± 0.35 ^b^	2.38 ± 0.29 ^b^	2.36 ± 0.30 ^b^	2.38 ± 0.05 ^b^	2.93 ± 0.52 ^a^	2.08 ± 0.34 ^b^	3.28 ± 0.48 ^a^	2.02 ± 0.53 ^b^
**Phenols**									
**guaiacol**	-	4.38 ± 0.14 ^b^	3.43 ± 0.35 ^d^	4.53 ± 0.55 ^b^	6.88 ± 0.72 ^a^	4.19 ± 0.73 ^b,e^	5.34 ± 0.51 ^b^	6.09 ± 0.05 ^a^	3.30 ± 0.70 ^d,e^
**Terpenes**									
**limonene**	33.83 ± 1.33 ^a^	8.72 ± 0.54 ^b^	2.99 ± 0.09 ^d^	2.61 ± 0.10 ^e^	4.78 ± 0.53 ^c^	3.08 ± 0.39 ^d^	3.01 ± 0.35 ^d^	3.38 ± 0.61 ^d^	1.80 ± 0.77 ^e^
**γ-terpinene**	5.76 ± 0.11								
**linalool oxide**	17.79 ± 0.38 ^a^	1.16 ± 0.09 ^f^	0.99 ± 0.03 ^f^	1.79 ± 0.12 ^e^	1.73 ± 0.24 ^e^	5.36 ± 0.26 ^c^	1.69 ± 0.14 ^e^	5.98 ± 0.16 ^b^	2.81 ± 0.64 ^d^
**α-terpinolene**	48.79 ± 0.47 ^a^	1.67 ± 0.32 ^b^	0.92 ± 0.14 ^d,e^	1.20 ± 0.05 ^c^	1.13 ± 0.16 ^c^	0.91 ± 0.003 ^d^	0.94 ± 0.06 ^d^	0.84 ± 0.16 ^e^	0.51 ± 0.13 ^f^
**linalool**	418.56 ± 17.99 ^a^	44.22 ± 1.52 ^c^	31.70 ± 1.18 ^d,f^	31.64 ± 4.89 ^d,f^	57.52 ± 3.28 ^b^	29.53 ± 0.60 ^d,f^	24.46 ± 3.46 ^e^	26.97 ± 3.78 ^e,f^	19.17 ± 1.34 ^g^
**menthol**	-	4.94 ± 0.11 ^a,c^	6.23 ± 0.31 ^a^	4.58 ± 0.71 ^a,b^	4.40 ± 0.66 ^b^	4.01 ± 0.27 ^b^	6.66 ± 1.67 ^a^	5.10 ± 0.03 ^a^	4.20 ± 0.85 ^b^
**α-terpineol**	374.22 ± 28.12 ^a^	22.27 ± 3.26 ^b,d^	17.33 ± 1.85 ^c^	24.16 ± 4.70 ^b,d^	28.05 ± 3.91 ^b^	19.59 ± 1.30 ^c^	23.24 ± 1.96 ^b^	19.63 ± 1.23 ^c,d^	12.98 ± 1.31 ^e^
**myrtenol**	35.23 ± 2.12 ^a^	5.62 ± 0.22 ^b^	3.00 ± 0.60 ^e^	4.96 ± 0.16 ^c^	3.13 ± 0.46 ^e^	4.19 ± 0.31 ^d^	2.20 ± 1.46 ^e,f^	2.85 ± 0.02 ^e^	2.02 ± 0.07 ^f^
**nerol**	34.69 ± 0.75 ^a^	2.41 ± 0.002 ^c^	1.15 ± 0.03 ^f,g^	1.78 ± 0.29 ^e^	3.21 ± 0.20 ^b^	1.59 ± 0.43 ^e,g^	1.64 ± 0.18 ^e^	2.03 ± 0.19 ^d^	1.18 ± 0.17 ^f,g^
**geraniol**	118.55 ± 3.39 ^a^	8.47 ± 0.53 ^c^	6.23 ± 1.24 ^d,e^	7.23 ± 0.72 ^c,d^	9.90 ± 0.63 ^b^	5.31 ± 0.27 ^e^	4.57 ± 0.28 ^f^	6.27 ± 0.81 ^d^	3.59 ± 0.63 ^f^
**vitispirane**	16.67 ± 0.14	-						-	
**α-ionol**	362.34 ± 31.83 ^a^	18.01 ± 0.80 ^c^	14.60 ± 1.07 ^d^	23.53 ± 3.88 ^b^	19.75 ± 1.51 ^b,c^	16.53 ± 0.82 ^c^	17.29 ± 2.70 ^c^	14.66 ± 0.16 ^d^	12.68 ± 1.19 ^e^
**β-damascenone**	38.74 ± 1.81 ^a^	2.72 ± 0.02 ^c^	1.96 ± 0.22 ^d,e^	3.08 ± 0.49 ^b^	2.65 ± 0.47 ^c^	2.02 ± 0.03 ^d^	2.36 ± 0.44 ^c,d^	2.24 ± 0.27 ^c,d^	1.59 ± 0.36 ^e^
***trans*-caryophyllene**	36.80 ± 1.27	-							
**α-ionone**	339.30 ± 8.32 ^a^	29.90 ± 0.67 ^b^	14.96 ± 1.71 ^d^	21.70 ± 2.61 ^c^	21.62 ± 1.43 ^c^	18.63 ± 2.79 ^c^	12.92 ± 0.95 ^d^	18.74 ± 2.16 ^c^	10.75 ± 0.02 ^e^
**dihydro-β-ionone**	152.40 ± 2.12 ^a^	26.13 ± 1.40 ^b^	11.32 ± 1.49 ^d,e^	17.32 ± 1.99 ^c^	12.61 ± 2.80 ^d^	13.30 ± 2.12 ^d^	9.51 ± 1.15 ^e^	13.97 ± 1.69 ^d^	6.85 ± 1.37 ^f^
**dihydro-β-ionol**	23.91 ± 0.12 ^a^	3.75 ± 0.09 ^b^	3.89 ± 0.81 ^b^	3.28 ± 0.37 ^b^	3.72 ± 0.56 ^b^	3.93 ± 0.52 ^b^	2.73 ± 0.04 ^c^	3.84 ± 0.61 ^b^	2.63 ± 0.36 ^c^
**β-ionone**	365.89 ± 20.27 ^a^	45.90 ± 0.20 ^b^	25.75 ± 4.60 ^d,e^	35.20 ± 4.35 ^c^	33.98 ± 6.29 ^c^	29.64 ± 3.75 ^c,d^	24.45 ± 2.85 ^d,e^	32.31 ± 4.07 ^c^	21.91 ± 0.44 ^e^
**myristicine**	8.51 ± 0.15	-						-	
**α-cedrol**	-	2.89 ± 0.20 ^b^	5.03 ± 1.02 ^a^	4.06 ± 0.19 ^a^	2.76 ± 0.40 ^b^	2.52 ± 0.002 ^b^	2.98 ± 0.53 ^b,c^	4.36 ± 0.84 ^a^	3.87 ± 0.60 ^a,c^

Within the row, means followed by superscript different letters are significantly different at *p* ≤ 0.05 (ANOVA, Fisher’s LSD).

**Table 3:** Volatile compounds (µg/kg) of cellulose/raspberry complexes prepared by 15 min and 60 min of complexation (2.5%, 5%, 7.5% and 10%—amounts of used cellulose) after storage.

Compounds	2.5%		5%		7.5%		10%	
Time of Complexation	15	60	15	60	15	60	15	60
**Aldehydes**								
**2-hexenal**	-	-	-	-		-		-
**benzaldehyde**	4.45 ± 0.03 ^e^	4.77 ± 0.47 ^d^	6.33 ± 0.01 ^a^	5.22 ± 0.07 ^c^	5.08 ± 0.11 ^c,d^	4.89 ± 0.20 ^d^	5.48 ± 0.09 ^b^	4.69 ± 0.17 ^d^
**octanal**	2.44 ± 0.02 ^b^	1.49 ± 0.11 ^e^	1.91 ± 0.01 ^c^	2.46 ± 0.06 ^b^	2.81 ± 0.12 ^a^	2.80 ± 0.16 ^a^	3.01 ± 0.14 ^a^	1.74 ± 0.01 ^d^
**nonanal**	7.00 ± 0.05 ^f^	6.37 ± 0.35 ^g^	13.94 ± 0.08 ^a^	8.60 ± 0.25 ^d^	8.05 ± 0.17 ^e^	9.73 ± 0.33 ^c^	12.57 ± 0.24 ^b^	9.11 ± 0.42 ^c,d^
**2-nonenal**	0.94 ± 0.01 ^c^	0.53 ± 0.05 ^e^	1.94 ± 0.01 ^a^	0.91 ± 0.02 ^c^	1.26 ± 0.04 ^b^	0.81 ± 0.00 ^d^	0.99 ± 0.01 ^c^	0.95 ± 0.08 ^c^
**decanal**	7.00 ± 0.01 ^d^	20.85 ± 0.66 ^a^	13.78 ± 1.42 ^b^	4.41 ± 0.17 ^e^	20.93 ± 0.99 ^a^	4.10 ± 0.24 ^e^	20.65 ± 0.75 ^a^	7.61 ± 0.21 ^c^
**ethyl benzaldehyde**	0.99 ± 0.01 ^d^	2.28 ± 0.20 ^a^	1.73 ± 0.01 ^b^	0.92 ± 0.08 ^d^	2.52 ± 0.02 ^a^	1.07 ± 0.12 ^d^	2.57 ± 0.12 ^a^	1.30 ± 0.01 ^c^
**4-propyl benzaldehyde**	6.72 ± 0.04^e^	10.23 ± 0.01 ^d^	11.82 ± 0.37 ^c^	12.45 ± 0.15 ^b^	10.68 ± 0.21 ^d^	13.66 ± 1.23 ^b^	11.68 ± 0.26 ^c^	15.39 ± 0.43 ^a^
**lilial**	-	2.14 ± 0.09 ^d^	-	2.64 ± 0.16 ^c^	-	3.11 ± 0.06 ^b^	-	3.35 ± 0.04 ^a^
**hexyl cinnamaldehyde**	1.18 ± 0.01 ^e^	1.60 ± 0.22 ^c,d^	1.38 ± 0.09 ^d^	1.37 ± 0.04 ^d^	1.73 ± 0.06 ^c^	2.13 ± 0.19 ^b^	2.30 ± 0.08 ^b^	2.57 ± 0.03 ^a^
**Ketones**								
**1-phenylethanone**	1.10 ± 0.03	-	-	-	-	-	-	-
**geranyl acetone**	4.43 ± 0.20 ^d^	5.14 ± 0.67 ^c^	5.57 ± 0.03 ^c^	3.22 ± 0.16 ^e^	4.81 ± 0.09 ^d^	5.49 ± 0.04 ^c^	7.49 ± 0.30 ^a^	6.22 ± 0.14 ^b^
**benzophenone**	1.10 ± 0.04 ^c^	1.45 ± 0.06 ^b^	1.45 ± 0.08 ^b^	1.32 ± 0.08 ^b^	1.79 ± 0.01 ^b^	2.63 ± 0.34 ^a^	2.55 ± 0.05 ^a^	2.30 ± 0.10 ^a^
**Esters**								
**methyl dihydrojasmonate**	1.44 ± 0.13 ^g^	2.48 ± 0.00 ^d^	2.08 ± 0.05 ^e^	2.37 ± 0.02 ^d^	1.84 ± 0.14 ^f^	3.04 ± 0.03 ^b^	4.83 ± 0.09 ^a^	2.71 ± 0.03 ^c^
**hexyl salicylate**	2.52 ± 0.05 ^c^	2.51 ± 0.16 ^c^	2.45 ± 0.01 ^c^	2.32 ± 0.14 ^c^	1.99 ± 0.01 ^d^	2.70 ± 0.24 ^b^	3.80 ± 0.16 ^s^	2.83 ± 0.11 ^b^
**Acids**								
**ethyl hexanoic acid**	1.10 ± 0.02 ^b^	^-^	0.59 ± 0.00 ^c^	^-^	1.57 ± 0.11 ^a^	^-^	1.15 ± 0.07 ^b^	-
**nonanoic acid**	1.19 ± 0.10 ^e^	1.52 ± 0.09 ^c^	1.66 ± 0.04 ^c^	6.03 ± 0.07 ^a^	1.02 ± 0.03 ^e^	1.79 ± 0.21 ^c^	2.45 ± 0.15 ^b^	1.34 ± 0.07 ^d^
**Alcohols**								
**2-ethyl hexanol**	21.48 ± 0.46 ^e^	19.40 ± 0.64 ^f^	30.78 ± 0.04 ^b^	20.69 ± 0.51 ^e,f^	32.06 ± 0.18 ^a^	23.92 ± 0.42 ^d^	26.00 ± 0.48 ^c^	17.27 ± 0.14 ^g^
**decanol**	1.18 ± 0.01 ^f^	1.45 ± 0.05 ^d^	1.48 ± 0.03 ^d^	1.18 ± 0.25 ^d,f^	1.73 ± 0.02 ^c^	1.27 ± 0.20 ^d,f^	2.36 ± 0.06 ^s^	1.94 ± 0.08 ^b^
**Phenols**								
**guaiacol**	2.29 ± 0.02 ^e^	2.40 ± 0.08 ^d^	2.19 ± 0.00 ^f^	2.89 ± 0.19 ^c^	3.53 ± 0.01 ^b^	3.83 ± 0.12 ^a^	3.72 ± 0.06 ^a^	2.56 ± 0.11 ^c,d^
**Terpenes**								
**limonene**	1.36 ± 0.00 ^d^	1.57 ± 0.07 ^c^	1.50 ± 0.04 ^c^	2.63 ± 0.07 ^b^	2.65 ± 0.13 ^b^	1.17 ± 0.22 ^d,e^	3.80 ± 0.05 ^a^	0.96 ± 0.01 ^e^
**linalool oxide**	1.26 ± 0.01 ^d^	0.99 ± 0.05 ^e^	2.09 ± 0.04 ^a^	1.43 ± 0.05 ^c^	1.84 ± 0.02 ^b^	1.55 ± 0.07 ^c^	1.29 ± 0.06 ^d^	1.53 ± 0.07 ^c^
**α-terpinolene**	0.64 ± 0.01 ^a,b^	0.73 ± 0.07 ^a^	0.77 ± 0.00 ^a^	0.75 ± 0.04 ^a^	0.57 ± 0.03 ^b^	0.67 ± 0.02 ^a,b^	0.71 ± 0.00 ^a^	0.62 ± 0.03 ^b^
**linalool**	20.75 ± 0.38 ^d^	24.59 ± 0.41 ^a^	23.61 ± 0.16 ^b^	26.78 ± 2.89 ^a^	24.94 ± 1.06 ^a^	22.20 ± 0.69^c^	25.64 ± 0.10 ^a^	20.71 ± 2.30 ^bd^
**menthol**	2.84 ± 0.14 ^e^	5.31 ± 0.45 ^b^	2.87 ± 0.09 ^e^	3.95 ± 0.01 ^c^	2.25 ± 0.00 ^f^	7.64 ± 0.39 ^a^	3.20 ± 0.12 ^d^	5.44 ± 0.35 ^b^
**α-terpineol**	15.67 ± 0.65 ^c^	16.98 ± 1.30 ^b,c^	17.32 ± 0.42 ^c^	19.99 ± 0.39 ^a^	18.32 ± 0.11 ^b^	20.36 ± 0.82 ^a^	20.02 ± 0.53 ^a^	18.38 ± 0.02 ^b^
**myrtenol**	1.44 ± 0.02 ^c^	2.05 ± 0.24 ^a,b^	1.42 ± 0.04 ^c^	2.29 ± 0.24 ^a^	1.38 ± 0.08 ^c^	2.05 ± 0.15 ^a,b^	1.97 ± 0.07 ^b^	2.04 ± 0.00 ^a,b^
**nerol**	0.85 ± 0.00 ^d^	0.91 ± 0.11 ^c,d^	1.55 ± 0.03 ^a^	0.91 ± 0.12 ^c,d^	0.97 ± 0.00 ^c^	0.94 ± 0.05 ^c^	1.18 ± 0.08 ^b^	1.22 ± 0.08 ^b^
**geraniol**	2.64 ± 0.08 ^e^	3.05 ± 0.06 ^d^	3.35 ± 0.10 ^c^	3.14 ± 0.24 ^c,d^	3.73 ± 0.03 ^b^	2.07 ± 0.19 ^f^	4.21 ± 0.13 ^a^	2.65 ± 0.03 ^e^
**α-ionol**	8.96 ± 0.11 ^c^	5.87 ± 4.68 ^d^	11.81 ± 0.43 ^b^	12.29 ± 1.53 ^b^	11.64 ± 0.07 ^b^	14.94 ± 0.78 ^a^	14.92 ± 0.48 ^a^	15.62 ± 1.10 ^a^
**β-damascenone**	1.13 ± 0.04 ^d^	1.55 ± 0.01 ^a^	1.19 ± 0.05 ^d^	1.09 ± 0.12 ^d^	1.12 ± 0.04 ^d^	1.17 ± 0.04 ^d^	1.43 ± 0.04 ^b^	1.27 ± 0.05 ^c^
**α-ionone**	8.38 ± 0.11 ^c^	9.51 ± 0.63 ^b^	7.70 ± 0.04 ^d^	9.58 ± 0.40 ^b^	6.34 ± 0.10 ^f^	6.89 ± 0.10 ^e^	13.38 ± 0.88 ^a^	9.94 ± 0.47 ^b^
**dihydro-β-ionone**	4.85 ± 0.00 ^c^	6.95 ± 0.48 ^a^	3.50 ± 0.09 ^e^	2.95 ± 0.18 ^b^	3.94 ± 0.11 ^d^	3.04 ± 0.04 ^f^	6.15 ± 0.05 ^b^	4.75 ± 0.20 ^c^
**dihydro-β-ionol**	1.72 ± 0.01 ^e^	2.63 ± 0.16 ^b^	2.04 ± 0.04 ^d^	2.16 ± 0.00 ^c^	2.98 ± 0.08 ^a^	2.27 ± 0.06 ^c^	3.09 ± 0.24 ^a^	2.88 ± 0.15 ^a,b^
**β-ionone**	8.42 ± 0.11 ^d^	12.51 ± 0.65 ^b^	6.63 ± 0.07 ^e^	12.06 ± 0.96 ^b^	6.80 ± 0.01 ^e^	9.75 ± 0.64 ^c^	14.59 ± 0.79 ^a^	12.28 ± 0.94 ^b^
**α-cedrol**	2.21 ± 0.10 ^c^	3.15 ± 0.28 ^b^	1.94 ± 0.08 ^d^	1.48 ± 0.05 ^f^	2.18 ± 0.08 ^c^	1.77 ± 0.20 ^e^	4.01 ± 0.16 ^a^	3.01 ± 0.21 ^b^

Within the row, means followed by superscript different letters are significantly different at *p* ≤ 0.05 (ANOVA, Fisher’s LSD).
